# Online Gaming and Well-Being in the English Longitudinal Study of Ageing

**DOI:** 10.1093/geroni/igab046.461

**Published:** 2021-12-17

**Authors:** Pamela Almeida-Meza, Dorina Cadar, Andrew Steptoe, Carrie Ryan

**Affiliations:** University College London, London, England, United Kingdom

## Abstract

Play is considered an important contributor to healthy ageing. Using data from 3,067 participants aged 50+ from the English Longitudinal Study of Ageing, we explored online gaming assessed at wave 6 (2012/13) and quality-of-life, loneliness, and depression at wave 9 (2018/19). Covariates were age, sex, marital status, education, work status, depression, self-rated health, physical activity, smoking and alcohol consumption. We found that 22% of respondents engaged in gaming. Interaction analyses indicated that for younger individuals (<65 years), gaming predicted lower scores in the self-realization sub-scale of the quality-of-life scale in comparison to older gamers. Furthermore, there was a significant association between gaming and lower quality-of-life for widowed individuals only, particularly in terms of autonomy, self-realization, and pleasure. There were non-significant associations between gaming and loneliness and depression. Online gaming might be independently associated with lower levels of quality of life, especially for younger and widowed adults.

